# Corrigendum to: Global burden of heart failure: a comprehensive and updated review of epidemiology

**DOI:** 10.1093/cvr/cvad026

**Published:** 2023-02-09

This is a corrigendum to: Gianluigi Savarese, Peter Moritz Becher, Lars H Lund, Petar Seferovic, Giuseppe M C Rosano, Andrew J S Coats, Global burden of heart failure: a comprehensive and updated review of epidemiology, *Cardiovascular Research*, Volume 118, Issue 17, December 2022, Pages 3272–3287, https://doi.org/10.1093/cvr/cvac013

In the originally published version of this manuscript author Giuseppe M C Rosano was incorrectly listed with two affiliations. The correct single affiliation should have been “IRCCS San Raffaele Roma, Rome, Italy.”

The following section on Funding was also inadvertently omitted:

